# Effects of a Warm Foot-Bath

**Published:** 1884-04

**Authors:** 


### EFFECTS OF A WARM FOOT BATH.

IN ORDER to determine whether warm foot baths were of any value as a derivative in cerebral congestion, or as a means of inducing a congestion of the pelvic organs, Dr. Sciolcowsky recently made a number of observations, noting the changes of temperature in the outer auditory miatus, the axilla and the rectum. The duration of each foot bath was from fifteen to twenty minutes, and the temperature of the water was from 92° to 97°. He found that the temperature rose in the axilla and the external auditory meatus, while it fell in the rectum. The volume of the vessels in the upper extremities was decreased. There was an increase in the frequency of the pulse and in the blood pressure.

These observations showed that the warm foot bath diminishes the flow of blood to the abdominal and pelvic organs, while, at the same time there is an increased flow to the superficial parts. The foot bath, then, neither diminishes the hyperæmia of the brain and its envelopes, nor increases that of the pelvic organs.

In Alaska everything freezes solid by the middle of October The mercury in winter falls 55 degrees below zero, and often lower

